# Annual acknowledgement of manuscript reviewers

**DOI:** 10.1186/1471-2296-15-17

**Published:** 2014-01-29

**Authors:** Magdalena Morawska

**Affiliations:** 1 BioMed Central, Floor 6, 236 Gray's Inn Road, London WC1X 8HB, UK

## Abstract

**Contributing reviewers:**

The editors of *BMC Family Practice* would like to thank all of our reviewers who have contributed to the journal in Volume 14 (2013).

## 

Abdulbari Bener

Qatar

Achilia Morrow

USA

Akke Vellinga

Ireland

Alain Mercier

France

Albert Aynsley-Green

UK

Alfonso Otero Gonzalez

Spain

Alice Theadom

New Zealand

Alison Booth

Australia

Allen Dietrich

USA

Aloysius Niroshan Siriwardena

UK

Amanda Howe

UK

Amy Tan

Canada

Amy Matser

Netherlands

An De Sutter

Belgium

Ana Ruigomez

Spain

Andre Tylee

UK

Andrea Scaramuzza

Italy

Andrea E Williamson

UK

Andreas Klement

Germany

Andrew Farmer

UK

Andrew Stoddart

UK

Angela Meade

UK

Angela Brega

USA

Ann Van Den Bruel

UK

Ann O'Malley

USA

Ann Dorrit Guassora

Denmark

Anna Chisholm

UK

Anne Macfarlane

Ireland

Anne Walsh

Australia

Anne Kennedy

UK

Anne Karen Jenum

Norway

Anneloes Van Staa

Netherlands

Annika Anden

Sweden

Annunziata Faustini

Italy

Ans H Tiessen

Netherlands

Anthony Viera

USA

Anthony Scott

Australia

Antonis Kousoulis

UK

Antonius Schneider

Germany

Arash Rashidian

Iran

Athina Tatsioni

Greece

Barbara Michiels

Belgium

Barbara Griffin

Australia

Barbara Schouten

Netherlands

Barry Lambe

Ireland

Bart Van Den Eynden

Belgium

Baruch Itzhak

Israel

Ben Gray

New Zealand

Bengt Arnetz

USA

Berend Terluin

Netherlands

Beth Cummings

Canada

Birgitte Jjm Schoenmakers

Belgium

Bob Mash

South Africa

Bonnieca Islam

Canada

Borislav Dimitrov

UK

Brian Mckinstry

UK

Brona Fullen

Ireland

Bruno Rushforth

UK

Caleb Ferguson

Australia

Cara Litvin

USA

Carl Edvard Rudebeck

Sweden

Caroline Laurence

Australia

Caroline Mitchell

UK

Carolyn Chew-Graham

UK

Carolyn Ahlers-Schmidt

USA

Carolyn Thorpe

USA

Catherine Darker

Ireland

Catherine Hyde

UK

Catherine Walshe

UK

Cathy Mihalopoulos

Australia

Charilaos Lygidakis

Italy

Cheryl Hunter

UK

Chester Fox

USA

Chima Ndumele

USA

Christa Meisinger

Germany

Christiaan Lako

Netherlands

Christian De Mey

Germany

Christie Cabral

UK

Christina M Stapelfeldt

Denmark

Christoph Gerhard

Germany

Christopher Burton

UK

Christopher Carroll

UK

Christopher Dowrick

UK

Christopher Butler

UK

Christopher Harrison

UK

Christos Lionis

Greece

Christy Pu

Taiwan

Clare Jinks

UK

Concepcion Violan-Fors

Spain

Conor Teljeur

Ireland

Cristina Lopez-Del Burgo

Spain

Daniel Bereczki

Hungary

Danielle Hitch

Australia

Darinka Klancar

Slovenia

David Howard

USA

David Seamark

UK

David Coultas

USA

David Armstrong

UK

David Price

UK

David Smith

Australia

David Ekers

UK

David Pierce

Australia

David Mclain

USA

Davorka Vrdoljak

Croatia

Deborah Finfgeld-Connett

USA

Delyth James

UK

Demosthenes Panagiotakos

Greece

Dianne Delva

Canada

Donald Mack

USA

Dong Joon Kim

South Korea

Donna Southern

Australia

Donna Mak

Australia

Douglas Roblin

USA

Edward Post

USA

Eileen Britt

New Zealand

Eliane Perrin

Switzerland

Elizabeth Halcomb

Australia

Elizabeth Murray

UK

Elizabeth Dean

Canada

Elizabeth Cottrell

UK

Elizabeth Barley

UK

Elizeus Rutebemberwa

Uganda

Elspeth Wise

UK

Emiel Hoogendijk

Netherlands

Emma Clarke

UK

Enriqueta Pujol-Ribera

Spain

Ernest Vinyoles Bargalló

Spain

Esra Saatci

Turkey

Eva-Carin Lindgren

Sweden

Evelien De Schepper

Netherlands

Fahui Wang

USA

Fiona Moir

New Zealand

Fiona Walter

UK

Fiona Boland

Ireland

Foteini Anastasiou

Greece

France Légaré

Canada

Frances Drummond

Ireland

Francesc Orfila Pernas

Spain

Francesca Innocenti

Italy

Francesco Carelli

Italy

Frank Peters-Klimm

Germany

Frank Buntinx

Belgium

Frank Smeenk

Netherlands

Franka Meiland

Netherlands

Gemma Wallace

UK

Geoffrey Wall

USA

Gerard Bury

Ireland

Gerardo Moreno

USA

Gerdien Franx

Netherlands

Gerry Gormley

UK

Gill Harvey

UK

Gillian Caughey

Australia

Gillian Bartlett

Canada

Glenn Robert

UK

Grant Russell

Australia

Grete Moth

Denmark

Guido Miccinesi

Italy

Günther Egidi

Germany

Guy De Backer

Belgium

Gwyneth Rees

Australia

H.G.A. Jochemsen-Van Der Leeuw

Netherlands

Haleama Al Sabbah

Belgium

Hanna Kaduszkiewicz

Germany

Harm Van Marwijk

Netherlands

Hasse Melbye

Norway

Heike Hansen

Germany

Helen Croker

UK

Helen Mclachlan

Australia

Helena Britt

Australia

Helena Roig Carrera

Spain

Helene Verdoux

France

Helene Vaillant-Roussel

France

Henk Schers

Netherlands

Heribert Sattel

Germany

Holger Wahl

Germany

Horst Christian Vollmar

Germany

Ib Jacobsen

Denmark

Ilaria Tarricone

Italy

Ingmar Schaefer

Germany

Isabelle Scholl

Germany

Isobel Cameron

UK

Ivon Milder

Netherlands

Jacqui Young

Australia

James Graham

USA

Jan De Lepeleire

Belgium

Jan De Mol

Belgium

Jane Morrow

Australia

Jane Roberts

UK

Janet Hanley

UK

Janet Seeley

Uganda

Janet Lefroy

UK

Janka Koschack

Germany

Janneke Noordman

Netherlands

Jason Wang

USA

Javier Garcia-Lacalle

Spain

Jay Giri

USA

Jean Muris

Netherlands

Jean-Marie Degryse

Belgium

Jeannine Coreil

USA

Jeetesh Patel

UK

Jenny Doust

Australia

Jesse Crosson

USA

Jessie Vanswearingen

USA

Jo Hart

UK

Joanna Moschandreas

Greece

Joanne Turnbull

UK

Joanne Dalton

USA

Joe Moran

Ireland

Joerg Haasenritter

Germany

Johan Wens

Belgium

John Yaphe

Portugal

John Cleland

UK

John Furler

Australia

John Mckay

UK

John Howie

UK

John Robson

UK

John Cape

UK

John Heesakkers

Netherlands

Jon Dowell

UK

Jonathan Benn

UK

Jonathan Vangeest

USA

Jonathan Silverman

UK

Joni Bosch

USA

Jorgen Nexoe

Denmark

Joseph Azuri

Israel

Joseph Lockwood

UK

Joseph Kvedar

USA

Jothydev Kesavadev

India

Judith Cole

UK

Juha Teperi

Finland

Julia Mirsky

Israel

Julie Schmittdiel

USA

Julie Polisena

Canada

Julie Green

UK

Julie Mcdonald

Australia

Jutta Bleidorn

Germany

Karen Smith

UK

Karen Voigt

Germany

Karen Fairhurst

UK

Karen Hoare

New Zealand

Karlijn J Joling

Netherlands

Katarina Stavrikj

Macedonia

Kate O'Donnell

UK

Kate Steinbeck

Australia

Kate Jolly

UK

Katrina Turner

UK

Kay Jones

Australia

Kay Wilhelm

Australia

Kazuhiro Yoshiuchi

Japan

Kelvin Hill

Australia

Kelvin Lynn

New Zealand

Kirsten Berk

Netherlands

Kirsti Malterud

Norway

Koen Putman

Belgium

Konrad Schmidt

Germany

Kota Katanoda

Japan

Kurt Stange

USA

Leona Hakkaart-Van Roijen

Netherlands

Lars Peterson

USA

Lars Gelander

Sweden

László Kalabay

Hungary

Laurie Tomlinson

UK

Leah Avery

UK

Lei Lian

China

Leif Eriksson

Sweden

Lesley Smith

UK

Lesley Martine Roberts

UK

Lidewij Broekhuizen

Netherlands

Lieve Peremans

Belgium

Linda Ooms

Netherlands

Linda Gask

UK

Linda Penn

UK

Linda Mcgowan

UK

Lisa Horowitz

USA

Lisa Crossland

Australia

Lisa Meredith

USA

Liset Van Dijk

Netherlands

Lorraine Wallace

USA

Louise Robinson

UK

Louise Logan

UK

Louise Acheson

USA

Luciana Ballini

Italy

Lucio Naccarella

Australia

Lucy Yardley

UK

Lyndal Trevena

Australia

Lynn Gallagher-Ford

USA

Mahendra Patel

UK

Malin Tistad

Sweden

Marc Van Nuland

Belgium

Marcello Migliore

Italy

Marcus Hollander

Canada

Margaret Cupples

UK

Margaret Maxwell

UK

Margaret Gatti-Mays

USA

Margareta Westerbotn

Sweden

Margrethe Smidth

Denmark

Maria Papadakaki

Greece

Maria Rodriguez-Sanz

Spain

Maria Isabel Fernández San Martín

Spain

Marian Oud

Netherlands

Marie Wood

USA

Marie-Josee Fleury

Canada

Marijn Alice Prins

Netherlands

Marjan Van Den Akker

Netherlands

Marjan Faber

Netherlands

Mark Ashworth

UK

Mark Gabbay

UK

Mark Spigt

Netherlands

Mark Harris

Australia

Mark Viron

USA

Mark Murphy

Ireland

Mark Porcheret

UK

Mark Kelly

UK

Mark Massing

USA

Mark Ebell

USA

Marleen Smits

Netherlands

Marloes Gerrits

Netherlands

Marta Buszewicz

UK

Martin Schulz

Germany

Martin Bardsley

UK

Martina Kadmon

Germany

Martine Granek-Catarivas

Israel

Mary Palmer

USA

Mary Clarke

Ireland

Mary Cannon

Ireland

Mateja Bulc

Slovenia

Mathijs Lucassen

New Zealand

Matthew Mcgrail

Australia

Matthew Harris

UK

Matthias Briel

Switzerland

Mauro Laudicella

UK

Megan Elliott-Rudder

Australia

Merryn Gott

UK

Mette Bech Risør

Norway

Michael Green

Canada

Michael Goldstein

USA

Michael Moore

UK

Michael Pentzek

Germany

Michael Harris

UK

Michal Shani

Israel

Michelle Howard

Canada

Miguel Roman

Spain

Miguel Muñoz

Spain

Miguel Bedolla

USA

Miranda Laurant

Netherlands

Miriam Santer

UK

Mohammadreza Hojat

USA

Mohammed Alazri

Oman

Moira Stephens

Australia

Molly Byrne

Ireland

Monica Greco

UK

Mònica Monteagudo

Spain

Nadeem Qureshi

UK

Nat Wright

UK

Natalia Loskutova

USA

Neesha Patel

UK

Neil Campbell

UK

Neil Heron

UK

Nettie Blankenstein

Netherlands

Ngiap Chuan Tan

Singapore

Nicholas Glasgow

Australia

Nicholas Zwar

Australia

Nick Francis

UK

Nicole Fowler

USA

Nicole Koburger

Germany

Nicole De Zoysa

UK

Nik Sherina Hanafi

Malaysia

Nikki Van Dessel

Netherlands

Nils Schneider

Germany

Nobutaka Hirooka

Japan

Norbert Donner-Banzhoff

Germany

Onno Van Schayck

Netherlands

Pablo Alonso-Coello

Spain

Päivi Rautava

Finland

Pal Gulbrandsen

Netherlands

Pali Hungin

UK

Panagiotis Simos

Greece

Parker Magin

Australia

Patricia R Casey

Ireland

Patrick Moran

Ireland

Patrick O'Connor

USA

Paul Sinfield

UK

Paul Rutter

UK

Paul Van Royen

Belgium

Paul Thomas

UK

Paul Padfield

UK

Per Fink

Denmark

Peter Vedsted

Denmark

Peter Schmolck

Germany

Peter Murchie

UK

Peter Verhaak

Netherlands

Peter Bower

UK

Peter Alan Coventry

UK

Philip Wilson

UK

Pieter Van Den Hombergh

Netherlands

Pietro Ragni

Italy

Polona Selic

Slovenia

Quinti Foguet-Boreu

Spain

R Erandie Ediriweera De Silva

Sri Lanka

Rachael Wood

UK

Ralf Jendyk

Germany

Raliat Onatade

UK

Ralph Panos

USA

Ralph Stacey

UK

Raymond Roberge

USA

Rebekah Pratt

USA

Rhona Hogg

UK

Richard Kones

USA

Richard Baker

UK

Richard Morriss

UK

Richard Turner

Australia

Richard Birtwhistle

Canada

Richard Neal

UK

Risa Hayes

USA

Robert Mckinley

UK

Robert Ross

USA

Rodolfo Valdez

USA

Rosalind Adam

UK

Rosemary Ikem

Nigeria

Rufus Cartwright

UK

Rupert Payne

UK

Ruth Robertson

UK

Sabrina Wong

Canada

Samuel Searle

Canada

Sandra Capra

Australia

Sanjoti Parekh

Australia

Sara Willems

Belgium

Sara Muller

UK

Sara Macdonald

UK

Sarah Shih

USA

Sarah Alderson

UK

Sarah Yardley

UK

Sarah Drew

UK

Sarah Smithson

UK

Sarah Knowles

UK

Sarah Hudson Scholle

USA

Seng Kwing Cheong

Singapore

Sevinç Kutlutürkan

Turkey

Shamil Haroon

UK

Sherina Mohd Sidik

Malaysia

Shirley Alexander

Australia

Shlomo Vinker

Israel

Sibyl Anthierens

Belgium

Siebrig Schokker

Netherlands

Simon Crouch

Australia

Sina Waibel

Spain

Sinead Brophy

UK

Siw Carlfjord

Sweden

Sophia Eilat-Tsnani

Israel

Staffan Nilsson

Sweden

Stefan Bösner

Germany

Stefan Sauerland

Germany

Stefania Salmaso

Italy

Stephen Gillam

UK

Stephen Neville

New Zealand

Stephen Abbott

UK

Stephen Brunton

USA

Steve Macgillivray

UK

Steven Ornstein

USA

Stewart Mercer

UK

Stuart Anderson

UK

Sudeh Cheraghi-Sohi

UK

Sue Kirby

Australia

Sue Patterson

Australia

Suhaila Ghuloum

Qatar

Supasit Pannarunothai

Thailand

Susan Darlow

USA

Susan Kirk

UK

Susan Hrisos

UK

Susan Smith

Ireland

Susannah Mclean

UK

Swan Yeap

Malaysia

Takashi Arao

Japan

Tara Donker

Australia

Tarun Sen Gupta

Australia

Teresa Harrison

USA

Thomas Laws

Australia

Thomas Kuehlein

Germany

Thomas Kubiak

Germany

Tim Stokes

UK

Tim Usherwood

Australia

Tim Holt

UK

Tjard Schermer

Netherlands

Tobias Freund

Germany

Tom Love

New Zealand

Tom Fahey

Ireland

Tony Dowell

New Zealand

Ueli Bollag

Switzerland

Ugur Bilge

Turkey

Ulrike Rothe

Germany

Ulrike Junius-Walker

Germany

Valerie King

USA

Valerio Zacà

Italy

Verna Lee

Malaysia

Veronika Schoeb

Switzerland

Vishnu Madhok

UK

Vivian R Ramsden

Canada

Waris Qidwai

Pakistan

Whye Lian Cheah

Malaysia

Willem Odendaal

South Africa

Willemijn Schäfer

Netherlands

Winifred Paulis

Netherlands

Wolfgang Hoffmann

Germany

Xuerui Tan

China

Yvonne Forsell

Sweden

Zalika Klemenc-Ketis

Slovenia

Zeljko Reiner

Croatia

Zumin Shi

Australia

## Pre-publication history

The pre-publication history for this paper can be accessed here:

http://www.biomedcentral.com/1471-2296/15/17/prepub

